# Homoprejudiced violence among Chinese men who have sex with men: a cross-sectional analysis in Guangzhou, China

**DOI:** 10.1186/s12889-020-08540-9

**Published:** 2020-03-27

**Authors:** Dan Wu, Eileen Yang, Wenting Huang, Weiming Tang, Huifang Xu, Chuncheng Liu, Stefan Baral, Suzanne Day, Joseph D. Tucker

**Affiliations:** 1 International Diagnostics Centre, London School of Hygiene and Tropical Medicine, Keppel Street, Bloomsbury, London, WC1E 7HT UK; 2 Social Entrepreneurship to Spur Health (SESH) Global, Guangzhou, China; 3 Institute for Global Health and Infectious Diseases, University of North Carolina at Chapel Hill, Chapel Hill, North Carolina USA; 4 Behavioral Sciences and Health Education, Rollins School of Public Health, Emory University, Atlanta, GA USA; 5 University North Carolina at Chapel Hill, Project-China, Guangzhou, China; 6 Dermatology Hospital of Southern Medical University, Guangzhou, China; 7 Guangzhou Center of Diseases Control, Guangzhou, China; 8 Department of Sociology, University of California San Diego, San Diego, USA; 9 Department of Epidemiology, The Johns Hopkins School of Public Health, Baltimore, MD USA

**Keywords:** Epidemiology, Stigma, Homoprejudice, Violence, Men who have sex with men, China

## Abstract

**Background:**

Homoprejudiced violence, defined as physical, verbal, psychological and cyber aggression against others because of their actual or perceived sexual orientation, is an important public health issue. Most homoprejudiced violence research has been conducted in high-income countries. This study examined homoprejudiced violence among men who have sex with men (MSM) in Guangzhou, China.

**Methods:**

MSM in a large Chinese city, Guangzhou, completed an online survey. Data about experiencing and initiating homoprejudiced violence was collected. Multivariable logistic regression analyses, controlling for age, residence, occupation, heterosexual marriage, education and income, were carried out to explore associated factors.

**Results:**

A total of 777 responses were analyzed and most (64.9%) men were under the age of 30. Three-hundred-ninety-nine (51.4%) men experienced homoprejudiced violence and 205 (25.9%) men perpetrated homoprejudiced violence against others. Men who identified as heterosexual were less (AOR = 0.6, 95% CI: 0.4–0.9) likely to experience homoprejudiced violence compared to men who identified as gay. Men who experienced homoprejudiced violence were more likely to initiate homoprejudiced violence (AOR = 2.44, 95% CI: 1.6–3.5). Men who disclosed their sexual orientation to other people were more likely to experience homoprejudiced violence (AOR = 1.8, 95% CI:1.3–2.5).

**Conclusions:**

These findings suggest the importance of further research and the implementation of interventions focused on preventing and mitigating the effects of homoprejudiced violence among MSM in China.

## Background

Homoprejudiced violence is a major public health issue [1]. Homoprejudiced violence is defined as physical, verbal, psychological, and cyber aggression against an individual, group or community based on their actual or perceived sexual orientation [2–6]. Homoprejudiced violence can be directed at people who identify as sexual minorities or those who are perceived as being a sexual minority.

The United Nations (UN) has recognized homoprejudiced violence as an important public health problem [4]. Homoprejudiced violence can result in physical and psychological harm, decreased productivity, and increased risk of addictions (e.g., substance and alcohol use) [4, 6–9]. However, homoprejudiced violence is often under-reported because victims are afraid of disclosing their sexual orientation [10] and there are limited resources for survivors [9, 11].

There is less research on homoprejudice in low- and middle-income countries (LMICs) [12, 13], including China [4]. One Chinese study found that 40.7% of sexual minorities experienced name calling, 34.8% were verbally abused, 22.4% were isolated in school, and 6.0% received physical violence threats [6]. Furthermore, existing evidence is focused more broadly on sexual minorities as a whole [9]. Previous research on LGBT youth in the United States reported that gay men are at higher risk of ostracization and receiving homoprejudiced remarks compared to lesbian and bisexual subgroups [14, 15]. However, little is known about the experiences of homoprejudiced violence among gay men or other men who have sex with men (MSM). Discrimination and homoprejudiced violence are known to influence sexual orientation disclosure [7, 10, 16] and uptake of HIV and other sexual health services [17]. A study in the United States reported that homoprejudiced violence victimization during youth was associated with more condomless sex and higher risk of HIV during adulthood [18].

Homoprejudiced violence initiated by gay men has not been well studied [9]. Many people who do not conform to gender and sexual norms are stigmatized, especially among men [14]. Masculinity is a primary component of socially desirable gender expression for men, and according to Connell’s theory of hegemonic masculinity, aggression is a feature of masculinity [19, 20]. As a result, both heterosexual men and closeted gay men may act in an aggressive way towards gay people to demonstrate their masculinity and differentiate themselves from gay men. Some gay men are afraid of receiving homoprejudiced violence and in order to hide their sexual orientation, show aggressive behaviors against LGBT groups to reinforce their masculinity [6, 9]. Understanding homoprejudiced violence may help to improve resources and develop interventions. The purpose of this study was to examine the frequency and correlates of homoprejudiced violence among MSM in Guangzhou, China.

## Material and methods

### Online survey

In partnership with a local community-based organization (CBO) and the Guangzhou Center for Disease Control and Prevention (CDC), we conducted a cross-sectional online questionnaire survey with 777 MSM in Guangzhou, China in September 2018. The survey was distributed online to MSM through CBO and CDC social media accounts. Eligibility criteria included the following: being biologically male at birth; being 16 years old or above; reported ever having oral or anal sex with men; residing in Guangzhou in the past six months. All survey data were anonymous and confidential, and online consent was obtained prior to the survey. Each man who participated received either 7.5 USD (50 Chinese Yuan) or a free HIV self-test kit as an incentive to participate.

### Survey instruments

We collected information about participants’ sociodemographic characteristics including age, residence permit, occupation, heterosexual marital status (never married, engaged or married, and divorced/separated/widowed), annual income, highest education obtained (high school or less, some college, university, and postgraduate), gender identity (male, female, transgender, and unsure), sexual orientation (gay, bisexual, heterosexual, and unsure) and sexual orientation disclosure to people other than their partner(s) (yes/no).

### Homoprejudiced violence questionnaire

Twelve homoprejudiced violence survey items were designed based on previous literature [5, 21, 22]. We selected 12 items to cover four domains – physical assault, verbal aggression, psychological abuse, and cyber violence (Supplementary file 1). We translated and adapted the 12 items in order for them to be relevant to Chinese men. These 12 items asked whether a participant had ever experienced any of the following due to their sexual orientation: being gossiped about, being name called, being deliberately alienated or isolated, being threatened, being maliciously called gay, being spat on, having personal belongings damaged, being deprived of economic resources or personal belongings by someone (including family members), having personal freedom restricted by someone (including family members), being physically harmed (such as being slapped, beaten or kicked), being harmed on social media (such as WeChat and Weibo, the Chinese substitutes of WhatsApp and Twitter), and being harmed through phone calls or messages. The items were field tested with 10 participants and minor amendments were made for better clarity.

All 12 items used three responses: “yes”, “no” and “do not want to tell”. A new summative variable was generated by adding up the responses (“yes” were coded as 1, “no” or “do not want to tell” were coded as 0) of the 12 items to assess the overall prevalence. The summed value 0 was recoded as 0 (no prior experiences of homoprejudiced violence), and the summed values 1 to 12 were recoded as 1 (prior experiences of homoprejudiced violence of any type) (outcome 1). Additionally, one follow-up item asked whether participants had ever committed any of the 12 violent behaviors aforementioned against others due to their sexual orientation (yes, no, do not want to tell) (outcome 2). The Cronbach alpha value of the 12-item homoprejudiced violence questionnaire was 0.89.

### Data analysis

Descriptive analysis was used to describe sample characteristics, including sociodemographic backgrounds and frequencies of violence experiences. The two outcomes were dichotomized (with “no” and “do not want to tell” grouped together) in regression analyses. We conducted univariate and multivariable binary logistic regressions to examine sociodemographic factors associated with homoprejudiced violence. We reported odds ratios and 95% confidence intervals (CIs). Data were analyzed using SPSS, version 25.

## Results

**Fig. 1:**
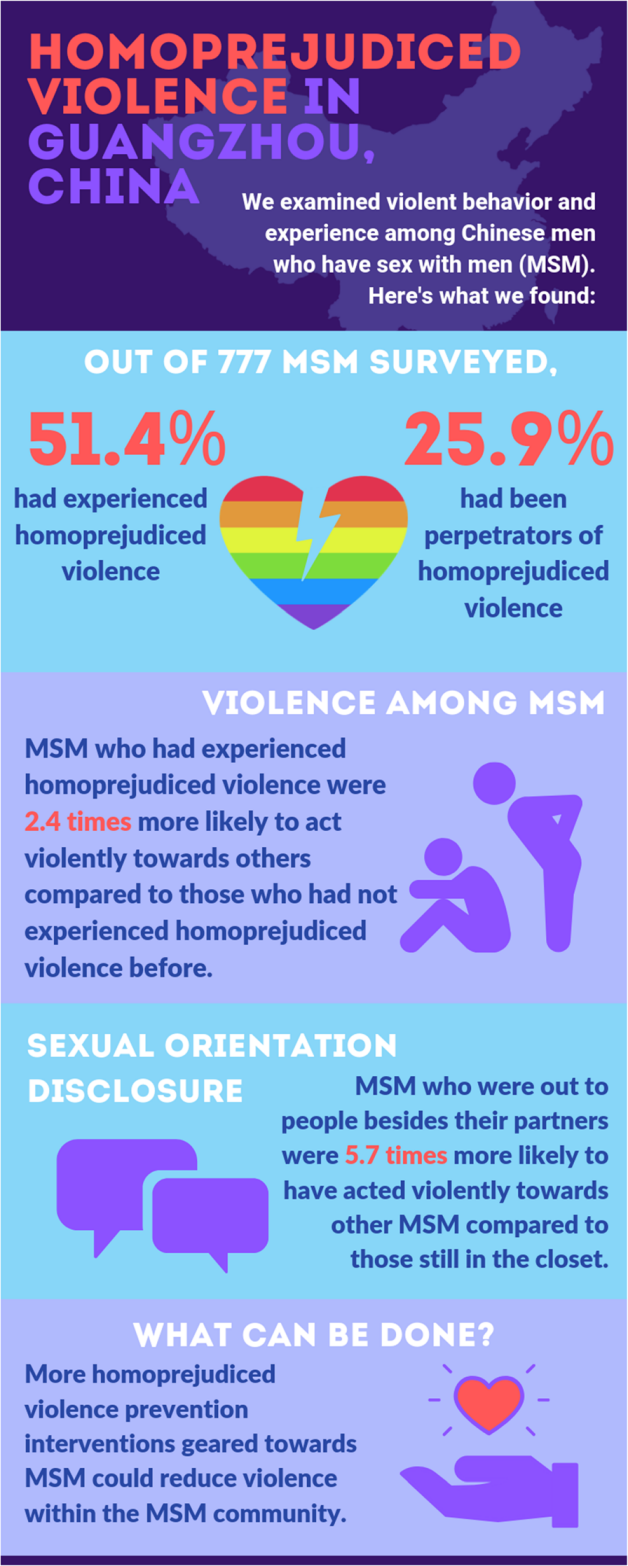
Infographic of homoprejudiced violence in Guangzhou, China. Source of data: The authors created this infographic based on the study findings by using a free graphic design website Canva (https://www.canva.com/)

We invited 2691 MSM to participate in the survey and 917 completed the questionnaire (response rate = 34%). Overall, 140 of these 917 MSM did not meet inclusion criteria and were excluded from the analysis. Data from 777 men were included in the analysis. Table 1 shows sociodemographic characteristics of the sample. Over half of survey respondents were under the age of 30 (495, 64.9%) and self-identified as gay (447, 57.5%). Most men lived in urban areas (639, 82.2%). A large proportion of men were not students (718, 92.4%), and about half had obtained university-level education or above (440, 56.7%). Around 40% (313) of men earned an annual income between US$8682–13,024, and nearly three-quarters had never been engaged or married to a woman (574, 73.9%). Most men had disclosed their sexual orientation to people other than their partners (571, 73.5%). A total of 399 (51.4%) men reported experiences of homoprejudiced violence, while 205 (25.9%) men initiated homoprejudiced violence (Fig. 1). Frequencies of each violence item are reported in Table 2. One hundred and six men (13.4%) experienced physical violence. One hundred and eighty-three men (23.1%) experienced name calling. Two hundred men (25.2%) experienced social isolation. One hundred and thirteen men (14.2%) experienced deprivation of economic resources or personal belongings and 128 (16.1%) reported cyber violence on social media.

**Table 1:**
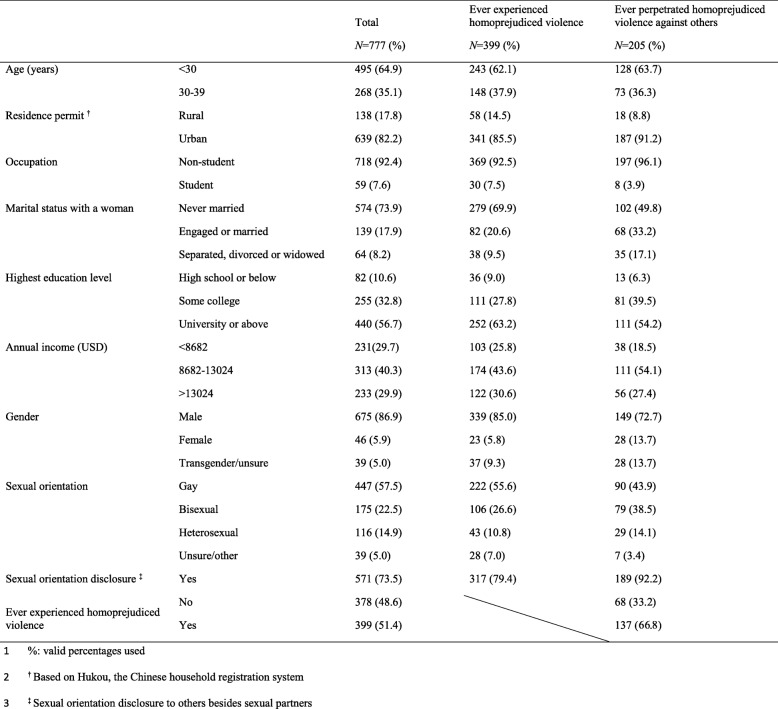
Sample characteristics of MSM who have experienced/engaged in homoprejudiced violence in Guangzhou, China in 2018 *(N = 777)*

**Table 2:** Frequency of individual homoprejudiced violence items n (%)

Individual items	Yes
1. Have you ever been gossiped due to your sexual orientation?	217 (27.4)
2. Have you ever experienced name calling due to your sexual orientation?	183 (23.1)
3. Have you ever been deliberately alienated or isolated due to your sexual orientation?	200 (25.2)
4. Have you ever been threatened due to your sexual orientation?	103 (13.0)
5. Have you ever been maliciously called gay due to your sexual orientation?	190 (24.0)
6. Have you ever been spat on due to your sexual orientation?	106 (13.4)
7. Did anyone damage your personal belongings due to your sexual orientation?	102 (12.9)
8. Have you ever been deprived of economic resources or personal belongings by anyone (including your family members) due to your sexual orientation?	113 (14.2)
9. Have you ever been restricted on personal freedom by anyone (including your family members) due to your sexual orientation?	98 (12.4)
10. Have you ever been physically harmed, such as be being slapped, beaten or kicked due to your sexual orientation?	106 (13.4)
11. Have you ever been harmed on social media (such as WeChat, Weibo) due to your sexual orientation?	128 (16.1)
12. Have you ever been harmed by phone call or messages due to your sexual orientation?	126 (15.9)
Total respondents who experienced any of the above violence	399 (51.4)

After controlling for demographic variables including age, residence status, occupation, marital status, education level, and annual income, multivariable logistic regression analyses showed that men who identified as heterosexual were less (AOR = 0.6, 95% CI: 0.4–0.9) likely to experience homoprejudiced violence compared to men who identified as gay.Men who were unsure about their sexual orientation were more likely (AOR = 2.6, 95% CI: 1.2–5.5) to have experienced homoprejudiced violence compared to gay men (Table 3). Men who disclosed their sexual orientation were,more likely (AOR = 1.8, 95% CI: 1.3–2.5) to experience homoprejudiced violence compared to other men (Table 3).

**Table 3:**
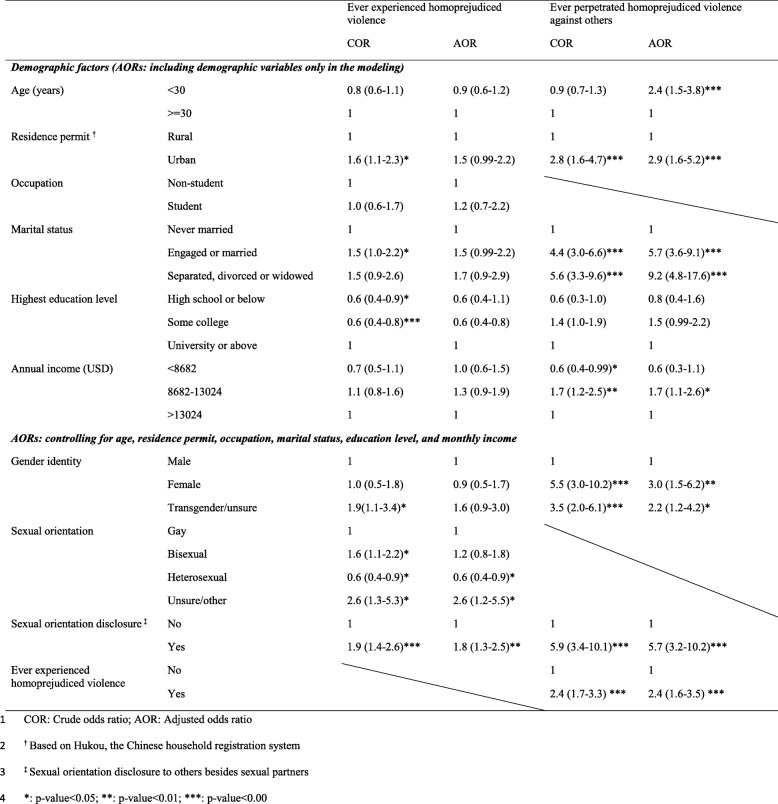
Factors associated with experiencing homoprejudiced violence/ever violating others among MSM in Guangzhou, China, in 2018 *(N = 777)*

Men younger than 30 years old were more likely to have initiated homoprejudiced violence against others compared to older men (AOR = 2.4, 95% CI: 1.5–3.8) (Table 3). Urban men were also more likely to have initiated homoprejudiced violence compared to rural men (AOR = 2.9, 95% CI: 1.6–5.2). Men who were engaged or married to women were more likely (AOR = 5.7, 95% CI: 3.6–9.1) to have initiated homoprejudiced violence than those who had never been married. Men who were separated, divorced, or widowed were more likely (AOR = 9.2, 95% CI: 4.8–17.6) to have initiated homoprejudiced violence compared to other men. People who identified as women or transgender/unsure were found to be 3.0 times (AOR = 3.0, 95% CI: 1.5–6.2) and 2.2 times (AOR = 2.2, 95% CI: 1.2–4.2) more likely to have been a perpetrator, respectively. Respondents who ever experienced homoprejudiced violence before were 2.4 times (AOR = 2.4, 95% CI: 1.6–3.5) more likely to have initiated homoprejudiced violence against others.

## Discussion

Homoprejudiced violence among sexual minorities is an important public health issue in many LMICs. Our study contributes to the literature by examining homoprejudiced violence among MSM in China, including MSM-initiated homoprejudiced violence .

We found that approximately half of men had ever experienced some form of homoprejudiced violence. This is lower than the prevalence of homoprejudiced violence observed in the UK [9] and US [11, 23]. Less homoprejudiced violence in Guangzhou may be related to lower levels of disclosing sexual orientation [24]. This finding is consistent with studies showing that more visible LGBT people may suffer from higher levels of violence [25, 26]. Interventions to reduce discrimination against sexual minorities are needed in China.

Many MSM in our sample who experienced homoprejudiced violence then went on to initiate homoprejudiced violence against other MSM. This finding is consistent with a study from the United States [27]. MSM may use violence as an approach to conceal their sexual orientation if they have been a victim of homoprejudiced violence [9]. Other potential factors may include poor sexual education and fear of social stigma. Poor awareness of homoprejudiced violence might also play a role. Understanding the context of homoprejudiced violence is key to successfully creating an environment where all MSM feel safe.

The data presented here have implications for research and policy. There are few epidemiological studies focusing on homoprejudiced violence among MSM in LMICs. Our study provides evidence on the prevalence and correlates of homoprejudiced violence. In terms of designing interventions, some subsets of MSM may be at greater risk for homoprejudiced violence. Our study suggests that younger, urban, and openly gay men are more likely to initiate homoprejudiced violence against others. Given young gay men are more often engaged in community-based sexual health programs, there may be missed opportunities for engaging community-based organizations to develop anti-violence interventions.

Our findings should be interpreted in the context of several limitations. First, we conducted the survey with MSM who subscribed to the social media account of a community-based organization that provided sexual health services in a developed city in China. Our participants had higher education and income than the average Guangzhou resident. Our study results should not be extrapolated to the wider community of MSM in China. Second, we combined participants’ responses of “no” and “don’t want to tell” to homoprejudiced violence as one category. This may result in a conservative or underestimate of the actual prevalence of homoprejudiced violence experiences due to unwillingness to share. Third, the study focused on homoprejudiced violence, but not broader experiences of homoprejudice. It is likely that non-violent experiences of homoprejudice among MSM are even more prevalent (e.g. social exclusion). Fourth, we recruited MSM who ever had anal or oral sex with a man in the study but did not include those who were gay men but had never engaged in sex with a man, limiting our understanding of the experiences of homoprejudiced violence to a subset of sexually active MSM. Lastly, an online cross-sectional questionnaire survey has limited depth to fully understand men’s thoughts about their own experiences. Qualitative research is warranted to better understand the issue.

## Conclusions

Homoprejudiced violence is an important public health problem. We found high a prevalence of homoprejudiced violence victimization and perpetration among Chinese MSM. Interventions are necessary to prevent homoprejudiced violence among Chinese MSM and create an environment where MSM feel safe.

## Supplementary information

**Additional file 1.** Homoprejudiced violence questionnaire.

## Data Availability

The datasets used and/or analyzed during the current study are available from the corresponding author on reasonable request.

## Abbreviations

MSM: Men who have sex with men
LMIC: Low- and middle-income countries
