# Supplementary material for: Homoprejudiced violence among Chinese men who have sex with men: a cross-sectional analysis in Guangzhou, China
Source: BMC Public Health. 2020 Mar 27;20:400. doi: 10.1186/s12889-020-08540-9 (PMC7099778; doi:10.1186/s12889-020-08540-9)
Supplement: Supplementary file 1 — Additional file 1. Homoprejudiced violence questionnaire. [file 12889_2020_8540_MOESM1_ESM.docx]

Supplementary file 1: Homoprejudiced violence questionnaire

| Individual items | Type of variable | Options |
| --- | --- | --- |
| 1. Have you ever been gossiped due to your sexual orientation? | Categorical | 1=Yes 2=No 3=I don't want to say |
| 1. Have you ever experienced name calling due to your sexual orientation? | Categorical | 1=Yes 2=No 3=I don't want to say |
| 1. Have you ever been deliberately alienated or isolated due to your sexual orientation? | Categorical | 1=Yes 2=No 3=I don't want to say |
| 1. Have you ever been threatened due to your sexual orientation? | Categorical | 1=Yes 2=No 3=I don't want to say |
| 1. Have you ever been maliciously called gay due to your sexual orientation? | Categorical | 1=Yes 2=No 3=I don't want to say |
| 1. Have you ever been spit on due to your sexual orientation? | Categorical | 1=Yes 2=No 3=I don't want to say |
| 1. Did anyone damage your personal belongings due to your sexual orientation? | Categorical | 1=Yes 2=No 3=I don't want to say |
| 1. Have you ever been deprived of economic resources or personal belongings by anyone (including your family members) due to your sexual orientation? | Categorical | 1=Yes 2=No 3=I don't want to say |
| 1. Have you ever been restricted on personal freedom by anyone (including your family members) due to your sexual orientation? | Categorical | 1=Yes 2=No 3=I don't want to say |
| 1. Have you ever been physically harmed, such as be being slapped, beaten or kicked due to your sexual orientation? | Categorical | 1=Yes 2=No 3=I don't want to say |
| 1. Have you ever been harmed on social media (such as wechat, Weibo) due to your sexual orientation? | Categorical | 1=Yes 2=No 3=I don't want to say |
| 1. Have you ever been harmed by phone call or messages due to your sexual orientation? | Categorical | 1=Yes 2=No 3=I don't want to say |
